# Recrafting the neighbor-joining method

**DOI:** 10.1186/1471-2105-7-29

**Published:** 2006-01-19

**Authors:** Thomas Mailund, Gerth S Brodal, Rolf Fagerberg, Christian NS Pedersen, Derek Phillips

**Affiliations:** 1 Bioinformatics Research Center (BiRC), University of Aarhus, Denmark; 2 Basic Research in Computer Sciences (BRICS), Department of Computer Science, University of Aarhus, Denmark; 3 Department of Mathematics and Computer Science, University of Southern Denmark, Odense, Denmark; 4 Department of Computer Science, University of Aarhus, Denmark; 5 School of Computer Science, University of Waterloo, Canada

## Abstract

**Background:**

The neighbor-joining method by Saitou and Nei is a widely used method for constructing phylogenetic trees. The formulation of the method gives rise to a canonical Θ(*n*^3^) algorithm upon which all existing implementations are based.

**Results:**

In this paper we present techniques for speeding up the canonical neighbor-joining method. Our algorithms construct the same phylogenetic trees as the canonical neighbor-joining method. The best-case running time of our algorithms are *O*(*n*^2^) but the worst-case remains *O*(*n*^3^). We empirically evaluate the performance of our algoritms on distance matrices obtained from the Pfam collection of alignments. The experiments indicate that the running time of our algorithms evolve as Θ(*n*^2^) on the examined instance collection. We also compare the running time with that of the QuickTree tool, a widely used efficient implementation of the canonical neighbor-joining method.

**Conclusion:**

The experiments show that our algorithms also yield a significant speed-up, already for medium sized instances.

## Background

The neighbor-joining method is a distance based method for constructing evolutionary trees. It was introduced by Saitou and Nei [1], and the running time was later improved by Studier and Keppler [2]. It has become a mainstay of phylogeny reconstruction, and is probably the most widely used distance based algorithm in practice. With a running time of *O*(*n*^3^) on *n *taxa [2], it is fast for small input, and empirical work shows it to be reasonable accurate, at least for cases where the rate of evolution is not extremely high or low. St. John et al. [3] even suggest it as a standard against which new phylogenetic methods should be evaluated. The aim of this paper is to improve on the running time of neighbor-joining tree reconstruction to make it applicable for larger datasets, e.g. [4]. Whether the accuracy supplied by the neighbor-joining method is useful for a particular data set in a particular situation is an independent issue outside of the scope of this paper.

The neighbor-joining method is a greedy algorithm which attempts to minimize the sum of all branch-lengths on the constructed phylogenetic tree. Conceptually, it starts out with a star-formed tree where each leaf corresponds to a species, and iteratively picks two nodes adjacent to the root and joins them by inserting a new node between the root and the two selected nodes. When joining nodes, the method selects the pair of nodes *i*, *j *that minimizes the branch-length sum of the resulting new tree. One way of achieving this [2] is always to select the pair of nodes *i*, *j *that minimizes

*Q*_*ij *_= (*r *- 2) *d*_*ij *_- (*R*_*i *_+ *R*_*j*_),     (1)

where *d*_*ij *_is the *distance *between nodes *i *and *j *(assumed symmetric, i.e., *d*_*ij *_= *d*_*ji*_), *R*_*k *_is the *row sum *over row *k *of the distance matrix: *R*_*k *_= ∑_*i *_*d*_*ik *_(where *i *ranges over all nodes adjacent to the root node), and *r *is the *remaining *number of nodes adjacent to the root. When nodes *i *and *j *are joined, they are replaced with a new node, *A*, with distance to a remaining node *k *given by

*d*_*Ak *_= (*d*_*ik *_+ *d*_*jk *_- *d*_*ij*_)/2.     (2)

This formulation of the neighbor-joining method gives rise to a canonical algorithm that performs a search for min_*i*,*j *_*Q*_*ij*_, using time *O*(*r*^2^), and joins *i *and *j*, using time *O*(*r*) to update *d*. This search and join is continued until only three nodes are adjacent to the root (i.e. for *n *- 3 joins where *n *is the total number of species). The total time complexity becomes *O*(*n*^3^), and the space complexity becomes *O*(*n*^2^) (for representing the distance matrix *d*). For further discussions of the neighbor-joining method, see e.g. [5-7].

In this paper, we present techniques for speeding up the canonical neighbor-joining algorithm. Our algorithms construct the same phylogenetic trees as the canonical algorithm, but attempt to reduce the search time for min_*i*,*j *_*Q*_*ij *_a quad-tree [8] built on top of the *Q *matrix, or on a matrix that approximates the *Q *matrix.

We evaluate the performance of our algorithms empirically on distance matrices obtained from the Pfam collection of alignments [9,10], and compare the running time with that of the QuickTree tool [11], a widely used efficient implementation of the canonical neighbor-joining algorithm, which previously was shown to run faster than the implementations in the CLUSTAL W, and PHYLIP packages, and faster than the BIONJ implementation of a variant of the neighbor-joining method. The results show that the presented algorithms can give a significant speed-up over the standard neighbor-joining method, already for moderately sized instances. Indeed, evidence is given that the running time of the best of our algorithms evolves as Θ(*n*^2^) on the examined instance collection, as opposed to Θ(*n*^3^) for QuickTree.

## Results and discussion

To evaluate the presented methods, we have implemented them in a tool, QuickJoin, available at [12]. For evaluating the performance of QuickJoin we have compared the QuickJoin tree creation with the canonical neighbor-joining tree creation method, as implemented in the tool QuickTree [11]. The QuickJoin program takes a distance matrix of the taxa for input, and produces a tree as output. The QuickTree tool, likewise, can take a distance matrix as input and produce a tree as output. Additionally, it can take a multiple alignment as input, and produce either a distance matrix or a tree as output. When comparing the running time of the two tools, we call both tools with a distance matrix as input.

The platform where the experiments were conducted was a Linux RedHat 8.0 kernel 2.4.18–19.7, Pentium 4 2.66 GHz, 512 KB cache, 1 GB ram, both the QuickJoin program and the QuickTree program was compiled using gcc/g++ 3.1.1 with optimization -O3. To measure the running time of the programs we used the GNU time tool, the time report is the user time obtained by the time -f %e option (wall-time in seconds). For QuickJoin we examine both the method based on a depth-first search with cutoffs and the method based on a priority queue search — see the Methods section for details. For QuickTree there is only one way of building trees.

### Results on Pfam data

The data used for the first experiment were protein sequence alignments taken from the Pfam database [9,10], and translated into distance matrices using QuickTree.

**Figure 1:**
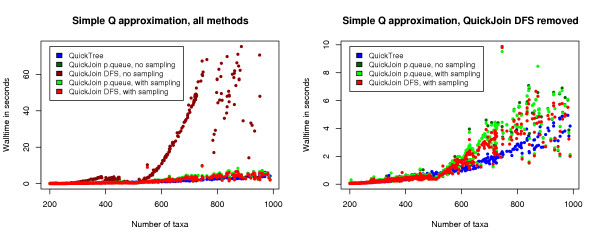
**Performance of our methods using the simple approximation to *Q***. The plots show the running time of QuickTree, and QuickJoin with the depth-first search (DFS) method and with the priority queue (p.queue) method with and without sampling (see Methods), with the first approximation of *Q *described in the Methods section. The input for the runs is distance matrices for the Pfam alignments with 200 to 1000 sequences. The depth-first search without sampling performs very poorly and is removed on the plot on the right to better show the performance of the remaining methods.

We first evaluated the performance of your method without the linear functions approximation of *Q *(see Methods). Figure 1 shows a plot of the walltime performance of the new methods with this approximation, compared to QuickTree on the alignments from Pfam with 200–1000 sequences. We can observe that the performance of the depth-first search method without sampling has a quite unstable performance, whereas the other methods achieve a performance comparable with that of the QuickTree implementation.

**Figure 2:**
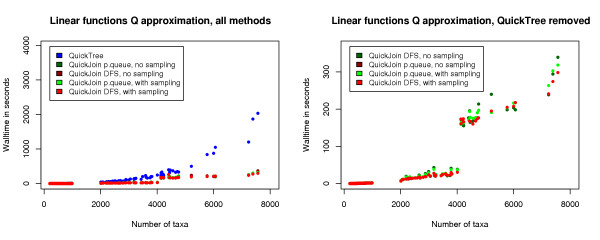
**Performance of our methods using the linear functions approximation to *Q***. The plots show the running time of QuickTree, and QuickJoin with the depth-first search method and with the priority queue method with and without sampling, with the linear functions approximation of *Q *described in the Methods section. The input for the runs is distance matrices for the Pfam alignments with 200 to 8000 sequences. The new methods perform significantly better than the basic neighbor-joining method, as implemented in QuickTree. To better compare the new methods the QuickTree plot is removed on the right.

We then evaluated the performance of the new methods when also using the linear functions approximation to *Q*. The input for the runs is distance matrices for the Pfam alignments with 200 to 8000 sequences, and the results are shown in Figure 2. We can observe that the running time of all the presented methods are at the same level, and that all the methods outperform the QuickTree implementation.

The way QuickJoin is implemented, the memory usage for representing the quad-tree is increased (by a factor of four) each time the number of taxa is increased to the next power of two. That is, the memory usage is close to constant between powers of twos, and grows by a factor of four when the input size crosses a power of two. As the memory usage grows, the number of page faults when running the program grows. This slows down the program, and is the explanation behind the increase in running time at 2^12 ^= 4096 in Figure 2. A similar increase in running time is observed at 2^11 ^= 2048 when running QuickJoin on a machine with less RAM. The canonical neighbor-joining method does not rely on a quad-tree and as such can run on less memory; it still needs to represent a distance matrix and a tree, however, and as such can only save about a factor of four compared to QuickJoin.

### Results on data provided by Georg Fuellen

We have also used QuickJoin on two datasets supplied by Georg Fuellen, Integrated Functional Genomics, University Hospital Muenster, who used neighbor-joining to produce large phylogenies as described in [4]. Dataset A is a multiple sequence alignment of 1138 species, and dataset B is a multiple sequence alignment of 1863 species. Both multiple sequence alignments were converted into corresponding distance matrices. Building trees using QuickTree took 8.29 sec for dataset A and 34.67 sec for dataset B. Building trees using QuickJoin took 3.09 (3.38) sec for dataset A and 6.50 (7.56) sec for dataset B when using the depth-first search (priority queue search) method.

## Conclusion

We have suggested methods for speeding up the search for min_*i*,*j *_*Q*_*ij *_in neighbor-joining based on a quad-tree storing information about known lower bounds on parts of the *Q *matrix. All our methods have a space bound of *O*(*n*^2^) and a time bound of the form *O*(*nS *+ *U*), where *S *is the time used (on average) in each search and *U *is the time used for updating and rebuilding the quad-tree and other auxiliary data structures. For the suggested methods, the update time has a worst case bound of *O*(*n*^2^) if we rebuild the quad-tree whenever we have halved the number of remaining nodes. A worst case bound for *S *is *O*(*n*^2^), resulting in a combined *O*(*n*^3^) time bound for the methods, i.e., the same asymptotic bound as the original method.

We have conducted experiments, evaluating the performance of the methods implemented in QuickJoin on data from the Pfam database and have shown that the methods perform favorably compared to the canonical algorithm as implemented in QuickTree and achieves a significant speed up. QuickTree is stated to be an optimized implementation of the Neighbor-Joining tree building algorithm [11]. We expect that if we apply a similar level of code optimization techniques to the implementation of QuickJoin used for the experiments we will be able achieve an improved performance increasing the gap between the performance of QuickTree and QuickJoin.

## Methods

Our algorithms construct the same phylogenetic trees as the canonical algorithm, but attempt to reduce the search time for min_*i*,*j *_*Q*_*ij*_, see Eq. (1), by using a quad-tree [8] built on top of the *Q *matrix, or on a matrix that approximates the *Q *matrix but does not need to be recomputed after each join. The nodes of the quad-tree store information guiding the search for the minimum, and the crux of our methods is to define this information in a way which will guide the search well for many iterations before it needs updating. The time complexity of our methods are given by *O*(*nS *+ *U*), where *S *is the average *search time *for finding nodes *i *and *j *minimizing *Q*_*ij*_, and *U *is the time used, throughout the algorithm, for *updating *the quad-tree and other bookkeeping information, e.g., the distance matrix. The worst case time complexity remains *O*(*n*^3^), but the anticipation is that our methods on real data is significantly faster. The space complexity after adding the quad-tree is still *O*(*n*^2^) since a quad-tree with *n*^2 ^leaves can be represented in *O*(*n*^2^) space.

### Using a quad-tree

**Figure 3:**
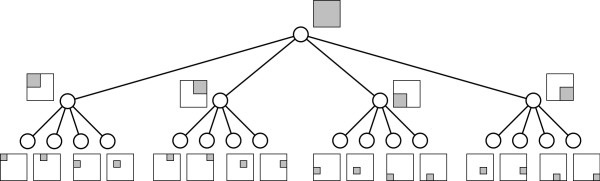
**A quad-tree with three levels of nodes**. A quad-tree with three levels of nodes, and the corresponding subdivision of a square. The root covers the entire square, its children each of the four quadrants, and the leaves a further division of these.

A quad-tree [8] is a four-ary tree modeling of a two-dimensional area recursively divided into quadrants. In the following description we assume for the sake of simplicity that *r *is a power of two. Figure 3 shows the tree resulting from a three-level recursive process.

By building a quad-tree of height log *r *— where *r *is the number of remaining neighbors to the root node — on top of the *r *× *r *matrix *Q *and storing in nodes of the quad-tree the minimal values in the subtree rooted at that node, we can search for the pair of nodes minimizing *Q*_*ij *_in time *O*(log *r*). However, by Eq. 1, in each iteration of the algorithm, all entries in *Q *need to be updated: the value *r *is decreased by one, and each row and column in *d *has a new distance to the joined node *A *added and two distances removed, thereby changing *R*_*k *_for all *k*. Updating *Q *after each iteration therefore takes time *O*(*r*^2^), leading to a running time of *O*(*n*^3^). There is no asymptotic gain, and in practice the quad-tree solution will be significantly slower than the basic, non-quad-tree, algorithm, as a consequence of the added overhead. Simply building a quad-tree on top of *Q *will not improve the running time.

The problem with building the quad-tree on top of *Q *is that all entries in *Q *change with each join. To decrease the update time, we need to build the quad-tree on some information that does not completely change with each join. If, for instance, we only need to update a single row and column per join, we can do that in *O*(*r*) time.

### Using approximations of *Q*

If we assume that the relative differences between the *Q*_*ij *_values do not change dramatically between joins — that is, we assume that the ordering of *Q*_*ij *_values is not randomly permuted after a join — we would expect that we could use the old *Q*_*ij *_values to guide the search for the current minimal *Q*_*ij*_. Let *Q' *denote the *Q *matrix at some earlier point, and let *r' *denote the number of remaining nodes adjacent to the root at that point. Similarly, let  denote the row sum of row *k *in *Q'*, and let *δ*_*k *_denote the difference between *R*_*k *_and : *R*_*k *_=  + *δ*_*k*_. Based on these definitions we can rewrite Eq. 1 to the following:

$Q_{ij}=\frac{r-2}{r'-2}Q_{ij}^{'}+\frac{r-r'}{r'-2}(R_{i}^{'}+R_{j}^{'})-(\delta_{i}+\delta_{j}).\;\left(3\right)$

This equation expresses the current *Q*_*ij *_values in terms of the old values and some *correction terms*, given by the *R' *and *δ *vectors. Because of these terms, the minimal  does not necessarily identify the nodes *i*, *j *that minimize *Q*_*ij*_, so we cannot use a quad-tree of *Q' *alone to find the nodes to join. We can, however, use a quad-tree over *Q' *to get *lower bounds *for the minimal *Q*_*ij *_value in parts of the *Q *matrix, as described in the following.

Let  denote a quad-tree built on top of *Q' *such that [*i*, *j*, *l*] denotes the minimum value at level *l*, where leaves are at level zero. More precisely, let [*i*, *j*, 0] = , and

$Q[i,j,l]=\min\{\begin{array}{l} Q[2i,2j,l-1]\\ Q[2i+1,2j,l-1]\\ Q[2i,2j+1,l-1]\\ Q[2i+1,2j+1,l-1]. \end{array}$

With this definition, we have

$Q[i,j,l]=\underset{2^{l}i\leq i'<2^{l}(i+1),\;2^{l}j\leq j^{'}<2^{l}(j+1)}{\min}Q_{i'j'}^{'}.$

Let  denote a binary tree for the correction terms built as follows, where [*k*, *l*] denotes the *k*th node at level *l*:

$\begin{array}{c} ℬ[k,0]=\frac{r-r^{\prime }}{r^{\prime }-2}{R^{\prime }}_{k}-\delta_{k}\\ ℬ[k,l]=\min\{ℬ[2k,l-1],\;ℬ[2k+1,l-1]\}. \end{array}$

We have

$ℬ[k,l]=\underset{2^{l}k\leq k'<2^{l}(k+1)}{\min}ℬ[k',0].$

>From the rewriting of *Q *by Eq. 3 and the trees above, we define

$ℒ[i,j,l]=\frac{r-2}{r'-2}Q[i,j,l]+ℬ[i,l]+ℬ[j,l].\;\left(4\right)$

We observe that [*i*, *j*, 0] = *Q*_*i*,*j *_and that [*i*, *j*, *l*] is a lower bound on the *Q *matrix entries in rows *2*^*l*^*i *to *2*^*l*^(*i *+ 1) - 1 and columns *2*^*l*^*j *to *2*^*l*^(*j *+ 1) - 1:

$ℒ[i,j,l]\leq \underset{2^{l}i\leq i'<2^{l}(i+1),\;2^{l}j\leq j'<2^{l}(j+1)}{\min}Q_{i'j'}$

The  values can be seen as a quad-tree, although it is implicitly defined by  and .

### Searching the quad-tree

We cannot simply search for the minimum valued leaf in  in the usual quad-tree search fashion, since we are no longer storing the minimum value in a range, but rather a lower bound on the minimum value. Instead, we will use the [*i*, *j*, *l*] values to guide our search for the minimal *Q*_*ij *_values.

Two approaches present themselves: A depth-first traversal of  with cut-offs when the lower bound is greater than a known *Q*_*ij *_value, and priority queue based search that always expands the [*i*, *j*, *l*] value with the lowest lower bound.

#### Depth-first search

In the depth-first search approach, we simply explore  in a depth-first manner, looking for the minimal [*i*, *j*, 0] value. By definition, this is also the minimal *Q*_*ij *_value. In itself, this will not speed up the search for the minimal value — although still in *O*(*r*^2^), traversing  is significantly slower than traversing the *Q *matrix to begin with — however, we can avoid exploring parts of the tree by cutting off searches of sub-trees. When we see a node [*i*, *j*, *l*], whose lower bound is greater than an already seen *Q *value at the bottom level, we need not explore the sub-tree rooted in [*i*, *j*, *l*] since none of the leaves in this tree will contain the minimal value.

The efficiency of this search greatly depends on how much of the tree can be discarded by cut-offs. In the worst case, no cut-offs are possible and we explore the entire , with a search time in *O*(*r*^2^), giving us a combined search time of *O*(*n*^3^). If, on average, we only need to explore *O*(*r*) nodes, the combined search time is down to *O*(*n*^2^).

#### Priority queue search

In the priority queue approach, we use a priority queue to expand the [*i*, *j*, *l*] nodes in a lowest-lower-bound-first order. This is based on the assumption that the lowest lower bound is more likely to contain the real lowest value. In each step, deletion of the minimum element in the priority queue gives us the unexplored node with the current lowest lower bound, and each of the children of the node are then inserted into the priority queue. Once a deletion produces an element on level 0, we have found the minimal *Q*_*ij *_value and need search no further.

As with the depth-first search, the efficiency of this search depends on how the lower bounds corresponds to the actual leaf-values in the tree. In the worst case, we need to explore the entire tree at a cost of *O*(*r*^2 ^log *r*), with a total search time of *O*(*n*^3 ^log *n*), while if, on average, we only search *O*(*r*) nodes we have a cost of *O*(*r *log *r*), with a total search time of *O*(*n*^2 ^log *n*).

#### Random sampling

Both the depth-first search and the priority queue search approaches can be extended with an initial random sampling of, e.g., *O*(*r*) entries of the *Q*_*ij *_matrix. The minimum of these values can then be used as the initial cut-off value. For the depth-first search approach this allows the algorithm to make more qualified cut-offs already from the beginning of the search, whereas for the priority queue search approach the gain is minimal since the cut-off only reduces the number of insertions into the priority queue — the number of deletions remains unchanged.

### Updating the quad-tree

In each join of two nodes, we need to delete two rows and two columns from *Q *— the two nodes we join — and add one new row and column — for the new node. This update must also be represented in , which means that we need to update  and . If we store the new row/column in one of the deleted rows/columns, say *i*, we need to update two rows/columns in *Q' *and all values in *δ *as follows (where  denotes the updated value of *x*):

$\begin{array}{c} \bar{d_{ik}}=(d_{ik}+d_{jk}-d_{ij})/2\\ \bar{\delta_{k}}=\delta_{k}+\bar{d_{ik}}-d_{ik}-d_{jk}\\ \bar{Q_{ik}^{'}}=(r'-2)\bar{d_{ik}}-(R_{i}^{'}+R_{k}^{'})\\ \bar{Q_{jk}^{'}}=\infty (\text{effectively deleting }j) \end{array}$

for all *k *≠ *i*, *j*. Updating *δ *and *Q' *this way takes time *O*(*r*). Rebuilding  from the new *δ *and updating  based on the change of two rows/columns in *Q' *can also be done in time *O*(*r*). Over the *n *iterations of the algorithm, this updating contributes *O*(*n*^2^) to the running time.

As the distance between *r *and *r' *grows, the information stored in *Q' *and *R'*, and thus in , diverges from the real values from *Q *and *R*. Consequently, the lower bounds in  becomes less accurate, and we expect to search more of  before we find a minimal leaf. It is therefore necessary to regularly update , by setting *Q' *to the current *Q*, updating *R' *and *δ *correspondingly, and rebuild .

A rebuild takes time *O*(*r*^2^), so if we rebuild too frequently, there will be no gain in running time — rebuilding in each iteration, for instance, will result in an *O*(*n*^3^) algorithm. On the other hand, if we rebuild too infrequently, the search time will suffer due to the worse lower bounds.

We chose to rebuild each time we have processed a fraction of the remaining nodes, i.e. after  iterations, for some fixed *m*. Since the size of the matrices constructed decreases exponentially, this implies that we spend *O*(*n*^2^) time on rebuilding all in all. Together with the updating performed in each iteration, this gives a total update time of *O*(*n*^2^).

### Limitations of the approach

For the methods to be useful, i.e. to yield a speed-up compared to a standard neighbor-joining implementation, it is essential that the derived lower bounds [*i*, *j*, *l*] for a node in the quad-tree are close to the minimum value among the leafs in the subtree spanned by the quad-tree node. Comparing Eq. 3 and Eq. 4 this might be infeasible if the correction terms

$\frac{r-r'}{r'-2}(R_{i}^{'}+R_{j}^{'})-(\delta_{i}+\delta_{j})$

span quite different values, since we use the minimum over all the correction values in the subtree.

Unfortunately, this is what we expect for the *R*_*i *_values. In our experiments presented in the Results section, we observe in Figure 1 that the performance of the above developed techniques, except for the depth first search without sampling, is essentially the same as those obtained by the QuickTree algorithm.

### Approximation of *Q *using Linear functions

In Eq. 3 we based our search on an old *Q *matrix. In this section we base the approach on the following rewriting of Eq. 1 that only depends on old row sums :

$\begin{array}{lll} Q_{ij} & = & \left(d_{ij}-\frac{R_{i}^{'}+R_{j}^{'}}{r'}\right)r\\ & & -2d_{ij}+\frac{rR_{i}^{'}}{r'}-R_{i}+\frac{rR_{j}^{'}}{r'}-R_{j}\\ & = & f_{ij}(r)+c_{i}(r)+c_{j}(r), \end{array}$

where

$f_{ij}(r)=\left(d_{ij}-\frac{{R^{\prime }}_{i}+{R^{\prime }}_{j}}{r^{\prime }}\right)r-2d_{ij}\;\left(5\right)$

$c_{i}(r)=r\frac{{R^{\prime }}_{i}}{r^{\prime }}-R_{i}.\;\left(6\right)$

The rewriting expresses *Q*_*ij *_as a linear function *f*_*ij *_over *r *plus some correction terms *c*_*i *_and *c*_*j *_— which, assuming  for *k *= *i*, *j*, is likely to be small. Note that *f*_*ij *_only depends on the current value of *d*_*ij *_and the values of *r' *and *R'*; we only need to update *f*_*ij *_when *i *or *j *is joined in a new node, i.e., we only need to update a linear number of functions for each join.

We will define below a quad-tree with the *f*_*ij *_functions at the leaves and where each internal node ideally should store the function that is the minimum over all the linear functions stored at the leaves of the subtree rooted at the node. Unfortunately this is not a linear function but a convex function consisting of piecewise linear functions. To achieve an efficient algorithm we instead, for the interval of *r *values of interest, maintain a lower bound for the convex function that is a linear function.

**Figure 4:**
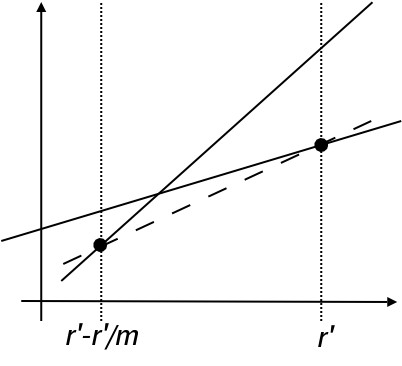
**The lower bound linear function**. A linear function that is the best lower bound of two other linear functions on the interval *r' *- *r'*/*m *to *r'*. The dashed line is the linear function that is the greatest lower bound of the two linear functions shown as solid lines.

Assume we decide to rebuild the structure after (at most)  iterations, for some fixed *m*. For two linear functions *f*_*ij *_and *f*_*i'j'*_, define min_*m*_{*f*_*ij*_, *f*_*i'j'*_} to be the linear function that passes through the two points

(*r' *- *r'*/*m*, min {*f*_*ij *_(*r' *- *r'/m*), *f*_*i'j' *_(*r' *- *r'*/*m*)})

and

(*r'*, min {*f*_*ij *_(*r'*), *f*_*i'j' *_(*r'*)}),

as illustrated by Figure 4. Defined this way, min_*m*_{*f*_*ij*_, *f*_*i'j'*_} is a lower bound for both of the functions until the next rebuilding:

min_*m*_{*f*_*ij*_, *f*_*i'j'*_} (*r*) ≤ min{*f*_*ij *_(*r*), *f*_*i'j' *_(*r*)},     (7)

for all *r *∊ [*r' *- *r'*/*m*, *r'*]. This minimum-operation is easily generalized to take the minimum of four functions, and we define a quad-tree  over the functions by:

[*i*, *j*, 0] = *f*_*ij *_(*r*)

$ℱ[i,j,l]=\min_{m}\{\begin{array}{ll} ℱ[2i,2j,l-1] & \\ ℱ[2i+1,2j,l-1] & \text{for }l>0\\ ℱ[2i,2j+1,l-1] & \\ ℱ[2i+1,2j+1,l-1] & \end{array}$

By induction on the number of minimum operations and a generalization of Eq. 7 we get

$ℱ[i,j,l](r)\leq \underset{2^{l}i\leq i'<2^{l}(i+1),\;2^{l}j\leq j'<2^{l}(j+1)}{\min}f_{i'j'}(r),$

for all *r *∊ [*r' *- *r'*/*m*, *r'*].

We can use this tree, together with a binary *correction tree * defined by

[*k*, 0] = *c*_*k*_(*r*)

[*k*, *l*] = min{[2*k*, *l *- 1], [2*k *+ 1, *l *- 1]} for *l *> 0

to define the implicit quad-tree

[*i*, *j*, *l*](*r*) = [*i*, *j*, *l*](*r*) + [*i*, *l*] + [*j*, *l*]

satisfying

$ℒ[i,j,l](r)\leq \underset{2^{l}i\leq i'<2^{l}(i+1),\;2^{l}j\leq j'<2^{l}(j+1)}{\min}Q_{ij}$

for the current *r*, assuming (*a*)  is updated along with the functions *f*_*ij *_whenever *i *or *j *is joined, (*b*) *r *∊ [*r' *- *r'*/*m*, *r'*], and (*c*)  is current. Condition *a *is necessary since *f*_*ij *_depends on *d*_*ij *_which changes when *i *or *j *is joined, and condition *b *is necessary because of the way the minimum operation is defined. Condition *c *simply states that since [*k*, 0] depend on the current value of *R*_*k*_, it must be updated whenever a join is performed.

#### Searching

Before each iteration  we must rebuild a current version of , which takes time *O*(*r*). After this, we can search for the minimal *Q*_*ij *_using the lower bounds in , as described in the previous section, using either a depth-first search with cut-offs or a priority queue. If the number of nodes visited during a search on average is linear, the total search time is *O*(*n*^2^) for the depth-first approach, or *O*(*n*^2 ^log *n*) for the priority queue approach.

#### Updating

For each join we must update two rows/columns in  taking time *O*(*r*) for a total of *O*(*n*^2^). Furthermore, we must completely rebuild the function tree  whenever *r *reaches *r' *- *r'*/*m*. For fixed *m*, this has a total cost of *O*(*n*^2^).

## Authors' contributions

TM implemented the algorithms in the QuickJoin tool. TM and RF conducted the experiments. All authors participated in the development of the algorithms, designing the experiments, and writing the paper.
